# Nurses on shacking ground—A qualitative study of Danish dermatology and allergology nurses' experiences of relocation during the COVID‐19 pandemic

**DOI:** 10.1002/nop2.2242

**Published:** 2024-07-11

**Authors:** Bettina Trettin, Nadja Trier Munk, Britt Egmose, Hanne Agerskov

**Affiliations:** ^1^ Department of Dermatology and Allergy Centre Odense University Hospital Odense Denmark; ^2^ Clinical Institute, Health Sciences University of Southern Denmark Odense Denmark; ^3^ Department of Nephrology Odense University Hospital Odense Denmark

**Keywords:** COVID‐19, E‐learning, nurses' experiences, qualitative study, relocation

## Abstract

**Aim:**

To investigate dermatology and allergology nurses' experiences of relocation from an outpatient clinic to a newly established COVID‐19 infectious disease ward.

**Design:**

A phenomenological‐hermeneutical approach was applied.

**Methods:**

Three focus groups with nurses were conducted from June to August 2020. Data were analysed in accordance with Ricoeur's theory of interpretation.

**Results:**

The relocation represented a challenging period that involved uncertainty and evoked feelings of excitement and dedication towards the nursing profession. Nurses felt obligated to help; however, they also experienced that they did not have a say in the relocation. The placement on the infectious disease ward was characterized by adaptations in three areas: unfamiliar working environment, unfamiliar team competencies and inadequate nursing training. E‐learning training was experienced as insufficient, as it did not enhance the nurses' specific competencies or confidence in caring for patients with COVID‐19.

**Conclusion:**

The relocation of nurses from an outpatient clinic to a new COVID‐19 infectious disease ward created a dilemma between nurses' sense of duty and their right to self‐determination. A prompt relocation into a newly established unfamiliar field caused frustrations because there were no unspoken rules to rely on. Managers should take nurses' experiences and perceptions under careful consideration and strive for more involvement in future scenarios.

**Patient or Public Contributions:**

No patient or public contribution.

## 1 INTRODUCTION

Having witnessed overload in healthcare systems in other parts of the world in connection with the COVID‐19 pandemic, the Danish government initiated a cutting edge plan to ensure sufficient capacity and facilities to provide optimal care and treatment for patients with COVID‐19. This placed an immediate demand on nurses, as the nurses in this study were promptly relocated from an outpatient clinic to a newly established infectious disease ward with staff from different disciplines and departments. Asynchronous e‐learning training programmes were developed to train and prepare the nurses for this task.

## 2 BACKGROUND

In March 2020, the World Health Organization characterized COVID‐19 as a pandemic. Since then, COVID‐19 has had a major impact on healthcare professionals' (HCPs) working conditions. Nurses, in particular, have played a pivotal role and been at the forefront of care of the pandemic since the beginning, as there was an immediate need to care for patients admitted with COVID‐19.

Both international and Danish national research indicates variation in how HCPs experienced the transfer to a different workplace and caring for patients with COVID‐19 (Thrysoee et al., 2022; Thude et al., 2021; Tort‐Nasarre et al., 2021). A crucial factor seemed to be whether the relocation was voluntary, or if the placement was mandatory (Thrysoee et al., 2022). Placement on a COVID‐19 infection ward resulted in feelings of inadequacy, powerlessness, fear, anxiety and distress (Fagerdahl et al., 2022; Joo & Liu, 2021; Specht et al., 2021; Thude et al., 2021). This was especially prominent among nurses employed at an outpatient clinic and among nurses who experienced that they had not volunteered to work on a COVID‐19 ward (Specht et al., 2021; Thrysoee et al., 2022).

Nurses who were unaccustomed to caring for patients with acute medical failure experienced a lack of training and guidelines in the management and care of patients with COVID‐19 (Joo & Liu, 2021; Thude et al., 2021; Tort‐Nasarre et al., 2021). While some experienced a ‘learning by doing’ culture (Tort‐Nasarre et al., 2021), others felt incapable of meeting the patients' complex care needs because they did not have the professional competencies (Sheng et al., 2020). Having access to new and validated knowledge about COVID‐19 was important to the nurses; however, the information flow was fast and not always delivered properly.

Nurses who had a visible, conscientious and facilitating manager felt more secure in their new working situation, because it helped them through an unpredictable new everyday life (Nowell et al., 2021; Thude et al., 2021). On the contrary, nurses who experienced an unreliable manager who failed to set an good example were more likely to experience anxiety and lack of confidence in their work capacity (Nowell et al., 2021).

While the qualitative research on nurses' experiences of caring for patients with COVID‐19 has been growing rapidly, very few studies have investigated the prompt relocation of nurses from outpatient clinics to newly established wards. Moreover, according to the research, nurses from outpatient clinics seemed to face different challenges, which points to the fact that education and training play a pivotal role. Thus, more in‐depth knowledge about what was experienced as essential is needed. Moreover, as an exception, the Department of Dermatology at a university hospital in Denmark was one of few departments in Denmark where nurses were employed *solely* at an outpatient clinic. Hence, they had no recent experience in caring for admitted patients and did not take part in shifts. Therefore, in‐depth knowledge of their experiences might represent an important contribution to the knowledge base, in order to learn how to manage future scenarios.

### 2.1 Research question

What were the experiences of dermatology and allergology nurses' of relocation to a newly established COVID‐19 infectious disease ward?

### 2.2 Design and method

This qualitative study used focus group interviews in order to explore the experiences and perspectives of the participants. A phenomenological‐hermeneutic approach was applied, inspired by Ricoeur's thoughts about narrative and interpretation (Ricoeur, 1976). To ensure and optimize the transparency of this study, the Consolidated Criteria for Reporting Qualitative Studies checklist guided the reporting of this study (Tong et al., 2007).

### 2.3 Sample and participants

In total, 12 dermatology and allergology nurses from an outpatient clinic were relocated during the pandemic. They were all invited to participate and included as eligible participants. The authors divided the participants into three focus groups, based on their knowledge of the participants' personalities, to ensure well‐functioning group dynamics and heterogenic groups, with the intention of maximizing the exploration of their various perspectives (Kitzinger, 1994). All participants were invited by email, and none declined to participate. Two focus group interviews were conducted 1 month after the relocation, in June 2020 and three months later, in August. At the time of the interview, 10 of the included nurses had returned to their usual workplace at the outpatient clinic. They were allocated for a period of 3 months. Two of the nurses were still working in the COVID‐19 infectious disease ward. Thus, all nurses had been involved in the care and management of critically ill patients who were tested PCR positive for COVID‐19. However, patients were admitted at an infectious disease ward and not an intensive care unit.

### 2.4 Data collection

The focus group interviews were conducted in a quiet conference room at the outpatient clinic using a semi‐structured interview guide. The interview guide was developed based on the (at that time) sparse, yet existing, research about nurses' experiences of caring for patients with COVID‐19 and nurses' experiences and perspectives of working on the frontline of a pandemic. As all of the participants were familiar to each other and to the researchers, the atmosphere during the focus group interviews was trusting. The interviews were conducted by the first author BT and BE was present as an observer, to take notes on the interaction and to ask questions to clarify issues raised, if necessary. During the focus group interviews, the participants were encouraged to reflect on and discuss their experiences and perspectives of being relocated to a newly established ward that cared for patients with COVID‐19. The overall question was how did you experiences the allocation from your occupation to the COVID‐19 ward? This was followed by prompts such as how did it make you feel? Please try to describe and discuss this. When investigating the e‐learning and training they had received during the relocation, the main question during focus group interviews was as follows: ‘How did you experience the teaching and training you received in connection to having to care for patients with COVID‐19?’ This was followed by promps such as ‘what was your greatest need in relation to training and education?’ and ‘Please, try to describe and discuss situations where you received training’. Finally, they were asked for their thoughts and perspectives about a possible new lockdown in the future and thus another possible relocation. A recent Danish research study had identified three archetypes of nurses caring for patients with COVID‐19 (Nielsen & Dieperink, 2020): the devoted, the noisemaker and the concerned. At the end of the focus group interviews, participants were asked to choose which of the three archetype they most identified with and to discuss their choice with each other. The focus group interviews lasted 105 min on average and were recorded and transcribed verbatim. Demographic information was collected using a short questionnaire, the questionnaire also asked about perceptions of the e‐learning training they had received during the period of their relocation, see Table 1.

**TABLE 1:** The background characteristics of the participants.

Participants	Age	Married/cohabitant	Number of home living children	Registered nurse y	Employed by outpatients clinic y	Experiences of infection medical department y	Years since last employment at a hospital department
A	40	Yes	2	16	5	None	9
B	36	Yes	2	13	4	8	4
C	59	Yes	0	27	8	None	19
D	57	Yes	0	31	11	12	12
E	39	Yes	2	14	8	6	6
F	42	Yes	2	13	5	None	6
G	43	Yes	2	21	7	None	7
H	39	Yes	2	14	7	None	12
I	39	Yes	2	16	8	None	8
J	40	Yes	2	15	5	None	5
K	39	Yes	2	14	8	None	8
L	38	Yes	2	14	6	None	6

### 2.5 Data analysis

The analysis was inspired by Ricoeur's theory of narrative and interpretation. Thus, it comprised of three levels: naïve reading, structural analysis and critical interpretation and discussion (Ricoeur, 1976). Data consisted of the focus group transcripts and questionnaire answers, which were gathered as one coherent text. The first level of the analysis, the naïve reading, was performed by way of reading the text several times. This was done to establish an initial impression of what the text was about. The naïve reading was written down, and this process was a step towards the first level of understanding. To obtain a deeper understanding about the participants' experiences and to validate the naïve reading, a structural analysis was performed. In this step, units of meaning (what is said) and units of significance (what the text speaks about) were identified. At this level, the transcripts were viewed objectively, to create distance from the text, thus, facilitating an analysis of the data as one whole text, in light of its content, and to create a new understanding (Ricoeur, 1973). In a dialectical process between explanation and understanding, themes emerged. Finally, to obtain an even deeper understanding of the findings, a subsequent critical interpretation and discussion was carried out, involving theory and other research results (Ricoeur, 1976). The analysis was carried out by BT and BE; however, findings were discussed among the entire research team.

### 2.6 Ethics

The study was approved by the Danish Data Protection Agency (20/26391) following the principles of the Declaration of Helsinki (World Medical Association, 2013). In accordance with Danish law, approval from the National Committee on Health Research Ethics was not required. All participants received oral and written information about the study and were told that they could withdraw from the study. Data were managed in accordance with the European Union's general data protection regulation (GDPR, 2016).

## 3 RESULTS

The naïve reading revealed that many different spectres of having to work in an unfamiliar department. Concerns about what was going to happen and how it would be to be relocated promptly. It seemed that there were difficulties in for the nurses to find out the criteria for choosing who was to be relocated and questions raised about justice and self‐determination. The relocation seemed to challenge both the nurses' professional competencies and their personal situations.

The structural analysis revealed four themes, which will be presented in the following section.

### 3.1 Feelings of obligation and dedication

The time leading up to the actual relocation involved expectations characterized by excitement, impatience and uncertainty:

I somehow had an expectation that something was about to happen, because we had been asked about our previous work experience. But the time until something actually did happen, it made me think: They might as well just take action now.

The uncertainty emanated from having heard about the overload of the healthcare systems in other parts of the world. It was a difficult task to be mentally prepared for a worst‐case scenario, and not knowing what to expect led to speculations and imaginings that aggravated feelings of uncertainty. The spectre of the forthcoming work situation included thoughts about the patients' severe illness conditions, fear of contagion, length of the relocation, collegial conditions and call for professional nursing competencies, which contributed to feelings of being on shacking ground. However, despite the uncertainty that characterized expectations and waiting time, there were feelings of devotion.

I felt a bit proud and thought, this is great, to be part of the covid situation and all that jazz. Then we can also say that we (as nurses in their department) have made a difference, we actually cared for these patients.

Feeling obligated was linked to experiences of the responsibility of being a nurse because there was an awareness about that they had the professional competencies to care for patients and to help out other colleagues by the virtue of their nursing education. Furthermore, they experienced that society in general put them under an obligation to act and take action. Thus, both societal and social expectations were related to feeling obligated and dedicated. At the same time, feelings of being proud arose and were linked to being able to not only help, but to make a significant difference during an ongoing pandemic. Hence, the nurses felt caught up in an emotional tension field between dedication and obligation.

### 3.2 Balancing the relocation, new working conditions and everyday life

The relocation had a major impact on the nurses' everyday lives. The government‐ordered lockdown affected the nurses' family rhythms, as they had to cope with missing childcare, home school teaching and their spouses also experienced a changed working life:

Doing shifts wasn't something that worked for me as a person, as a nurse or as a wife or mother. It is the reason I work at an outpatient clinic, because the working hours are extremely important to me. It was not particularly nice knowing that I couldn't opt out of it, I HAD to do it.

Having to find, achieve and adapt to new routines and to create a structure on an individual level was a constant effort in trying to balance the relocation and its conditions and its impact on family life. Furthermore, there were experiences of their families being concerned and because this was very important to the nurses, the obligation to help and being in a position where refusal was impossible caused a dilemma. Because the relocation was not time limited, it was at times experienced as incalculable and chaotic and demanded radical changes in everyday life.

The process of selecting nursing staff for relocation was experienced as involving a lack of transparency, because the nurses did not receive a reason or an explanation as to why they were relocated, while some of their colleagues were not:

You had to tell them (management) about your previous (work) experience, but no one told you anything about what that information was used for. I was on a list but didn't really know what was going to happen or what that meant. Why me?

The way the management obtained clarification about nursing competencies was perceived as unconnected and illogical. The missing information, for example of how the nurses were selected for relocation created speculation, insecurity and lack of confidence in the management. The nurses experienced feelings of being used as pawns in the strategy of recruiting staff for the new infectious disease ward. Questioning the decisions as to why they were relocated and others not was based on a search for some kind of justice; however, their search failed, which again led to speculation and new feelings of distrust. Thus, they were caught in a vicious circle that went beyond the relocation, as it led to speculation about repeated relocations in the future.

Having a choice or having a say in the relocation was experienced differently among the nurses. Some experienced not having a choice at all, while others thought that they would have had a choice if they had spoken up. However, they all reflected on and agreed that they did not have any leverage in the relocation and as a result a struggle for self‐determination occurred:

Being in a place (new ward) where under no circumstances I wanted to be. A place where in every way I had chosen not to be. Caring for patients with COVID‐19 was not difficult for me, but being on an in‐patient ward with a shift burden and all sorts of new colleagues, and never to know what to expect was hard.

Thus, trying to find a balance between the relocation, new working conditions and everyday life was experienced as chaotic, insecure with missing elements of involvement.

### 3.3 The complexity of training in a new working environment

The e‐learning training that the nurses had to complete did not contribute to the experience of being sufficiently prepared, more competent or familiar with caring for patients with COVID‐19:

And then this e‐learning popped up. In the beginning, I was quite effective and completed the courses, but eventually it just became lost in the crowd.

E‐learning was experienced as suitable for brush‐up of competencies; however, the number of e‐learning modules was extensive and over time became lost in the crowd of the extensive information flow of COVID‐19. There was a request for a structured and systematic approach to the training and a need for bedside training in clinical tasks; however, this was considered insufficient.

On arriving at the newly established ward, the nurses had no familiarity with the physical surroundings and experienced that there were no existing routines to rely on.

I think there was someone missing – a person who had the overview of what you needed to learn. In my opinion, no one took the lead.

There were requirements for someone to set an example and create a daily rhythm. There were experiences that no one took ownership or leadership in regard of the culture and daily rhythm on the ward. Therefore, the nurses had to feel their own way into how clinical and practical tasks were done, and when they were done.

The relocation created a rising awareness that several elements in the new work routine had pivotal influence on their working performance, both professionally and collegially:

You can't read everything in a book. The things it takes to work on a patient ward, the things that you only realize after many years and that no one speaks about. Why are you all going in that direction? You are turning to the right and I am standing right here thinking, why not turn left?

The spirit and culture on the newly established ward could not be taught using e‐learning or bedside training. The nurses emphasized that this was something that had to be learned through experiences, including tacit experiences. Thus, in particular, the complexity of training was followed by reflections on whether there was a culture, or whether there were any cultural rules to learn in a new work environment.

### 3.4 A challenging time of adaptation and adjustment

Being relocated as a group with colleagues they knew very well was of great importance, because it led to a sense of community, gave some peace of mind and made the collaboration with new colleagues easier:

In some ways, I felt excited because there was this special feeling of community spirit. We can really make this work when we have to.

The nurses attempted to adapt to and navigate in the new situation and to the surroundings, demands and work tasks, while they experienced insecurity regarding their professional skills:

It came to a point where my stomach hurt when the thought crossed my mind: could I, professionally, meet the requirements for caring for patients with COVID‐19? As a nurse it was demanding, I had to dig out skills that I hadn't used in years, which was quite anxiety‐triggering.

The feelings of dedication that were experienced prior to and at the beginning of the relocation faded out as time passed and frustrations arose. However, the nurses experienced that they had to establish a new ward within a new field/area of nursing care, with new routines, and a new culture, while rediscovering former competencies.

All at once we weren't the new ones anymore, then we could show them (new colleagues) the ropes and where to get their coffee. With time you got a sense of ownership, eventually you had a night shift on a ward that you were somehow familiar with.

Although it was a challenging time of adaptation and adjustment, over time a sense of community arose.

## 4 DISCUSSION

While e‐learning has been well investigated in nursing educational programmes and in clinical settings, e‐learning programmes with the aim of training and preparing nurses to care for patients with COVID‐19 are less characterized. We found that the number of e‐learning modules was complex and too extensive and thus became lost in the crowd because of a heavy information load. This is consistent with a study by Kong et al. (2021), who found that e‐learning at the beginning of the pandemic resulted in overwhelming information load, which negatively impacted HCPs' ability to recall information and led to information fatigue. While asynchronous e‐learning provides flexibility, its success also depends on the HCPs' motivation and self‐study (Uprichard, 2020). Thus, sustaining HCPs' engagement can be challenging and maybe especially when nurses also have to cope with the challenges of a relocation. In addition, we found that the nurses requested more bedside learning, which, combined with the fact that e‐learning training was experienced as insufficient, could indicate that the nurses had entered the new COVID‐19 ward unprepared. Lack of preparation can further increase both concerns and uncertainty among nurses (Danielis et al., 2021).

Our study found that, prior to the relocation, nurses did not know what to expect, which led to speculation and imaginings that aggravated feelings of uncertainty. These findings are similar to those of Sperling who, through the use of a qualitative questionnaire, investigated nurses' challenges and concerns during the COVID‐19 outbreak (Sperling, 2021). Sperling found that the most frequent themes of concern were related to work conditions, patients' interest, inter‐collegiate relationships and uncertainty and worries about the future. Our study further revealed that the relocated nurses felt caught up in an emotional tension field between dedication and obligation that created a dilemma. They found themselves in a position in which they felt both pressured to participate in and capable of participating in by the virtue of their education. Thus, our findings indicate that the nurses experienced a double commitment.

Another finding was the experience of lack of transparency in the process of selection for relocation, that is how the management obtained clarification about the nurses' competencies. This is somewhat consistent with Thrysoee et al. (2022), who found that creating positive experiences for nurses who were relocated led to feelings of being supported and safe, and this could be achieved through psychological security, practical support and the management's recognition of the nurses. Thus, being present, open and providing support and process transparency are important aspects for management to take into consideration in future scenarios. Furthermore, involving nurses in the process of the relocation, and allowing them to share their concerns may contribute to nurse feeling that they have a voice. This is particularly relevant, given that nurses across the world have experienced that their voices and perspectives were not included in COVID‐19 decision making. This is in spite of constituting the largest healthcare force, with direct and sustained contact with COVID‐19 patients throughout the pandemic (Rasmussen et al., 2022).

Being relocated to a newly established ward made the nurses realize how important it was to establish a culture or to be familiar with a culture. Thude et al. (2021) found that a barrier to coping with a relocation was lack of a sense of belonging, mostly linked to not receiving information or being familiar with practical situations. This is consistent with our findings, where not being part of an established culture likewise created a feeling of not belonging.

The Danish nurse and philosopher Merry Elisabeth Scheel, created the theory of interactional nursing practice, According to this theory, nursing practice is interactional and focuses on both theory and practice. The theory is based on three types of complementary knowledge and refers to Haberma's three modes of action: The cognitive‐instrumental, aesthetic‐expressive and moral‐practical. The cognitive‐instrumental mode of action aims to involve problem solving, result‐oriented activity and efficacy. The aesthetic‐expressive mode of action aims to understand a dialogue based on self‐reflection. This understanding is not only reserved to the individual patient's situation but also connected to the understanding and interaction among professionals. The moral‐practical mode aims to discuss action in relation to moral‐practical and legal issues, problems and task. Thus, the moral‐practical knowledge is a combination of ethical knowledge about how to create a relation to other people and knowledge of the practical situation, its possibilities and limitations (Nielsen et al., 2015; Scheel et al., 2008). According to Scheel's theory, the three modes of action should not be separated; instead, they should be considered as an inseparable whole. However, one mode may be more prominent, depending on the situation. In our study, we found that the nurses were assessed for selection based on their previous competencies, that they received e‐learning in an attempt to train and prepare them; however, the training lacked a systematic structure. The cognitive‐instrumental mode of action is, according to Scheel, focused, result oriented and efficient. Preparing nurses to strengthen only this mode of action may not have been enough. Furthermore, we found that, in the period before the relocation, the nurses sought an explanation as to why they had been chosen and questioned the justice in the decisions made. They felt like pawns and at the same time tried to balance a changed everyday life. Thus, the moral‐practical mode of action, which would focus on the nurses' overall situation, was not taken into account. The lack of transparency, dialogue and openness during the relocation process and the establishment of a culture they could thrive in challenged the aesthetic‐expressive mode of action. The findings of our current study call for attention on reasons why it may be important to align the three modes of action. Thus, to achieve enhanced nursing competencies, to motivate and create security among nurses, the focus should be on organizational and dialogue‐based individual conditions, as well as the ethical aspects, in relation to communication.

Our study found that the nurses had difficulties in adapting to their new surroundings, new colleagues and lack of direction in regard of how things should be done. They emphasized that it was something that had to be learned through experience, including tacit experiences. This finding has not been described elsewhere. According to the French sociologist Pierre Bourdieu, a field is characterized by doxa, which refers to the tacit knowledge or unspoken rules that exist, albeit are not voiced. The concept of doxa contains the specific rules for what is right and what is wrong in a certain field (Bourdieu, 1990). The rules are acknowledged by the individuals in the field and constitute its shared beliefs. With this theoretical aspect in mind, the nurses in our study were promptly relocated to a new, unfamiliar field and experienced frustrations as they discovered that there were no unspoken rules to rely on. According to Bourdieu and Wacquant (1992), a field is a network of relations that are based on the distribution of different kinds of capital. Capital can manifest itself in three forms: financial, cultural and social capital. Social capital refers to an individual's access to social networks and relationships and thus depends on social interaction. Norms, trust, reciprocity and obligation are factors that contribute to the aspects of social capital. Our findings point to the fact that the nurses experienced the relocation to be a challenging time and they initiated their new clinical tasks with no experience of being in possession of social capital. However, as the nurses became more familiar with the clinical tasks and other staff, they took on responsibility and, over time, a sense of community arose and they may have experienced a growth of social capital. This is an important aspect to address, because high levels of social capital among nurses have been associated with nurses' well‐being, retention, cooperation and patient safety (Read, 2014).

### 4.1 Limitations

The study was a single‐centre study, which is a limitation as it may question the transferability of the findings. However, the department where the study was conducted is the only dermatological department in Denmark with no in‐patient activity. All patients are treated at the outpatient clinic. Therefore, we considered that it offered a unique opportunity to investigate the experiences of the relocation of nurses working in this context. Furthermore, our study contributed with novel findings, for example the impact on nurses when they experienced not being able to grasp a sense of a culture. Another limitation is that three of the authors were employed at the department where the research was conducted, which may have influenced the research. According to Malterud (2001), it is inevitable not to affect the phenomenon under research when conducting a qualitative study. To avoid misinterpretation and over‐interpretation because of our preconceptions, the analysis was performed jointly by BT and BE, both experienced in qualitative research. Furthermore, the analysis was conducted systematically, reflected upon and discussed with the entire research team, which included a senior researcher with a different clinical background.

## 5 CONCLUSION

The relocation of nurses from an outpatient clinic to a new COVID‐19 infectious disease ward created a dilemma between nurses' sense of duty and their right to self‐determination. Even though inadequate qualifications were a concern, aspects such as teamwork and clinical working environment were highly significant E‐learning programmes were considered suitable for brushing up on basic nursing skills; however, updates on news, knowledge and research regarding COVID‐19 were missing and implementation of the programme called for more sufficient organization. A prompt relocation into a newly established, unfamiliar field causes frustrations because there were no unspoken rules to rely on. This lack of social capital may, with time, grow and contribute to a sense of community. Managers should take nurses' perceptions and experiences under careful consideration and strive for more involvement in future scenarios.

## AUTHOR CONTRIBUTIONS

Bettina Trettin, Nadja Trier Munk and Britt Egmose involved in study design. Hanne Agerskov has provided substantial contribution of the analysis and interpretation of findings. All authors have drafted the manuscript, and Hanne Agerskov has revised it critically for important intellectual content. All authors have agreed on the final version and meet at least one of the following criteria recommended by ICMJE.

## FUNDING INFORMATION

The Department of Dermatology and Allergy Centre, Odense University Hospital.

## CONFLICT OF INTEREST STATEMENT

The authors declare no conflicts of interest.

## Data Availability

Data is not publicly available but can be obtained from the authors for reason.
